# Prevalence of Stress, Depressive Symptoms, and Contributing Factors Among Undergraduate Nursing Students: A Systematic Review and Meta-Analysis

**DOI:** 10.3390/nursrep16030088

**Published:** 2026-03-05

**Authors:** Ling Xu, Michael Joshua Morales, Marianne Biangone, Thomas Hoffmann, Cherry Leung

**Affiliations:** 1 School of Nursing, University of California San Francisco, San Francisco, CA 94143, USA; marianne.biangone@ucsf.edu (M.B.); cherry.leung@ucsf.edu (C.L.); 2 Department of Veteran Affairs, San Francisco, CA 94121, USA; michaeljoshua.morales@va.gov; 3 School of Medicine, University of California San Francisco, San Francisco, CA 94143, USA; thomas.hoffmann@ucsf.edu

**Keywords:** stress, depression, nursing students, systematic review

## Abstract

**Background/Objectives**: Undergraduate nursing students experience high levels of stress and depressive symptoms. This study investigated the prevalence of stress and depressive symptoms among undergraduate nursing students and analyzed the sociodemographic and interpersonal factors influencing these conditions. **Methods**: Following the Preferred Reporting Items for Systematic Reviews and Meta-Analyses (PRISMA) guidelines, four databases (PubMed, Web of Science, Embase, and CINAHL) were searched for studies published between 2019 and 2024. Both narrative synthesis and meta-analysis were conducted to identify contributing factors and estimate the pooled prevalence rates. Prevalence rates of stress and depressive symptoms were estimated using random-effects models. Subgroup analyses were performed based on the severity of symptoms, study location, country income group, and measurement tool. Effect size, 95% confidence intervals, and *p*-values were reported. **Results**: The review included 54 studies. The prevalence of depressive symptoms and stress was 48% and 55%, respectively. Depressive symptoms were most prevalent in studies conducted in lower–middle-income countries and those using the Beck Depression Inventory II. Stress prevalence was highest in studies from upper–middle-income countries and those using the Perceived Stress Scale-10. Significant associations among sociodemographic and interpersonal factors include younger age, female gender, single status, financial issues, poor perceived health, conflicts with friends, and dissatisfaction with social activities. **Conclusions**: Nearly half of undergraduate nursing students from diverse settings experienced significant stress and depressive symptoms, driven by multiple sociodemographic and interpersonal factors.

## 1. Introduction

Undergraduate nursing students experience high levels of psychological stress (referred to as stress) and depressive symptoms. Stress refers to feelings of emotional strain or being overwhelmed when perceived demands exceed one’s coping abilities [1]. Depressive symptoms encompass a spectrum of feelings that include loss of interest, hopelessness, sleep disturbances, fatigue, poor appetite, difficulty concentrating, and self-harming thoughts [2]. Recent studies indicate that 19.5% to 42.1% of nursing students experience moderate to severe levels of stress [3], while 23% to 54% report moderate to severe depressive symptoms [4,5]. Further, elevated stress and depressive symptoms among undergraduate nursing students are associated with a range of adverse outcomes. These symptoms have been associated with lower academic performance, reduced clinical competency, and diminished professional commitment [6,7,8]. Behaviorally and physiologically, stress and depressive symptoms have been associated with substance use, disordered eating, sleep disturbances, and pain [9,10]. These broad impacts highlight the urgency of identifying the factors that place nursing students at greatest risk for psychological distress.

While academic [11] and clinical training [12,13] demands are well-established sources of stress among nursing students with implications on depressive symptoms, these educational stressors do not fully explain students’ broader psychological vulnerability. The contribution of sociodemographic and interpersonal factors to students’ distress remains less clearly understood. Prior studies suggest that younger age, female gender, single status, country of birth, and lower-income background are associated with elevated stress and depressive symptoms [14,15]. Interpersonal factors are defined as the reciprocal social and emotional interactions that individuals have with others in their environment. These include relationships with family, friends, romantic partners, colleagues, and members of the broader community, both within and outside the academic setting [16]. Such factors have been reported to exacerbate psychological challenges. For example, nursing students frequently encounter complex interactions with faculty, clinical staff, patients, and classmates, which contribute to their stress during clinical training [11]. Additionally, studies have identified broader social and relational concerns, including unsatisfactory family or peer relationships, experiences of discrimination, and feelings of loneliness as significant contributors to stress and depressive symptoms [17,18]. Elevated stress and depressive symptoms can increase the risk of burnout and suicidal ideation [19,20]. Therefore, understanding the factors that place students at risk is essential for developing effective interventions that support student well-being and professional success.

While previous systematic reviews have examined the role of demographic factors, including age, gender, year of study, and geographic location, on nursing students’ stress levels [3] and depressive symptoms [21], no review to date has comprehensively evaluated a broader range of sociodemographic or interpersonal factors. These include financial status, encompassing income level and financial difficulties; family situation, including living arrangements, family types, housing security; personal health, covering both physical and mental well-being; and interpersonal stressors, including social isolation, discrimination, and relationship challenges, with stress and depressive symptoms. This gap is particularly pressing in light of data from the 2018 American Psychological Association, which reported that Generation Z (typically individuals under age 25) frequently experiences interpersonal stressors such as perceived discrimination, social isolation, and relationship challenges [22]. Given that most undergraduate nursing students belong to this generational cohort [23], understanding the impact of these interpersonal stressors is critical.

To address these gaps, it is important to explore the range of sociodemographic and interpersonal factors that contribute to stress and depressive symptoms. It is also essential to assess the current prevalence of these mental health challenges among undergraduate nursing students, especially given the ongoing shifts in higher education, clinical training environments, and broader societal stressors affecting this population. Prevalence data offer essential insights into the scope and urgency of these conditions within nursing education today, helping identify which students are most at risk and why. These insights enable nursing programs to develop timely, evidence-informed interventions that support student mental health, enhance academic performance, improve retention, and prepare future nurses for the demands of an increasingly complex healthcare system. Ultimately, such efforts contribute to cultivating a resilient, diverse, and sustainable nursing workforce prepared to meet global healthcare challenges. This study aimed to (1) determine the prevalence of stress and depressive symptoms among undergraduate nursing students through a meta-analysis, and (2) examine the sociodemographic and interpersonal factors contributing to these outcomes using narrative synthesis.

## 2. Materials and Methods

### 2.1. Design

This study is a systematic review that includes both narrative synthesis and meta-analysis, which adheres to the Preferred Reporting Items for Systematic Reviews and Meta-Analyses (PRISMA) guidelines [24].

### 2.2. Search Methods

Two reviewers searched four databases—PubMed, Web of Science, Embase, and CINAHL—to identify studies on stress and depressive symptoms among undergraduate nursing students. The search included keywords such as nursing students, stress, depression, sociological factors, food security, housing, discrimination, loneliness, family relations, and friends, using various keyword combinations; see Table 1 for detailed search strategy by database. We limited inclusion to studies published between 2019 and 2024 to focus on contemporary prevalence and correlates of stress and depressive symptoms among undergraduate nursing students. This time frame was selected to capture research conducted within the more recent educational, technological, and sociocultural context of nursing training, recognizing that publication year serves as an approximate indicator of contemporary study conditions. Our aim was to reflect recent patterns rather than provide historical estimates across earlier decades. The review team conducted the final search on 6 June 2024.

The inclusion criteria were: (a) quantitative studies involving undergraduate nursing students or participants enrolled in pre-licensure nursing programs equivalent to the undergraduate level (e.g., BSN, vocational/diploma students), (b) studies examining the association between sociodemographic or interpersonal factors and stress or depressive symptoms, (c) studies reporting prevalence of stress or depressive symptoms or providing sufficient data to calculate prevalence, and (d) full-text articles published in English.

The exclusion criteria were: (a) studies not focused on undergraduate nursing students (e.g., licensed nurses, graduate students), (b) qualitative studies, (c) studies that provided neither prevalence data nor analyses of sociodemographic or interpersonal factors, (d) non-peer-reviewed studies, (e) studies examining COVID-19–related (pandemic-specific) stress or depressive symptoms, (f) theoretical works, (g) conference papers, (h) dissertations, (i) opinion pieces, (j) editorials, (k) review studies, and (l) study protocols.

### 2.3. Study Selection

Two reviewers independently evaluated the search results, removing duplicates using the Covidence software [25]. Articles were first screened by title and abstract based on the inclusion criteria. Full-text articles were then reviewed independently. Each reviewer documented exclusion reasons at both screening stages. Discrepancies were resolved through discussion.

### 2.4. Data Extraction Process

Two reviewers independently extracted data using the Covidence software. Before complete data extraction, the review team conducted a pilot test to ensure consistency and clarity in the extraction process. The review team discussed the types of data to be extracted and collaboratively developed a standardized extraction table. Both reviewers independently extracted data from a small subset of articles and compared results to assess consistency and refine the extraction form as needed. Following this pilot test, the review team performed independent extraction for all included studies. Any discrepancies in extracted data were resolved through discussion within the review team.

### 2.5. Data Item

Relevant data were extracted from eligible studies. This included the first author, study location, publication year, study design, sample characteristics, sample size, prevalence of stress or depressive symptoms, assessment instruments, and sociodemographic and interpersonal factors. Stress outcomes reflected general perceived stress and depressive symptoms focused exclusively on symptom severity assessed with validated screening scales (e.g., DASS-21, PHQ-9, BDI-II [26]). Clinical diagnoses, attachment-related distress, and other distinct psychological constructs were not included.

Sociodemographic variables included age, gender, race/ethnicity, marital status, academic year, geographic location, financial status, employment, income, number of children, living arrangement, parental education, food security, housing security, psychotropic drug use, mental health, and physical health. Interpersonal factors encompassed social relationships (family, friends, significant others, community support), loneliness, and discrimination. Quantitative outcomes and statistical data were also extracted, including significance tests (*p*-values), odds ratios, beta coefficients, confidence intervals, correlations, and 2 × 2 frequency tables.

### 2.6. Quality of Appraisal

Two reviewers independently conducted the quality assessment using the National Institutes of Health quality assessment tool for cohort, observational, or cross-sectional studies [27]. This tool comprises 14 items, each rated as “yes,” “no,” or “other” (not applicable; not reported). The assessment evaluated internal validity, including sample size justification, exposure measurement, outcome assessment, and the validity and reliability of measurement tools. Studies were categorized as “good,” “fair,” or “poor” based on their overall quality rating. Items rated as not applicable (NA) were excluded from the quality rating. Discrepancies in quality assessment scores were resolved through discussion and consensus between the reviewers [24].

### 2.7. Data Synthesis

#### 2.7.1. Narrative Synthesis Approach 

We conducted a narrative synthesis to summarize study characteristics, measurement approaches, and quantitative findings that could not be pooled for statistical analysis. Specifically, studies were summarized narratively when (1) the outcome was assessed using instruments that were not sufficiently comparable for pooling (e.g., limited psychometric documentation or not comparable to a commonly used tool), (2) associated factors were reported using inconsistent effect metrics (e.g., odds ratios vs. correlations vs. mean differences), or (3) studies used different categorizations of a variable (e.g., definitions of financial hardship). In all cases, general study characteristics were synthesized narratively, with tables and text summarizing study results and providing explanations [24].

Findings on contributing factors were summarized narratively in tables and text, as no studies were eligible for data pooling. Meta-analysis of associated factors requires that at least two studies reported the same factor using comparable definitions, effect metrics, instruments, and cut-offs [28]. Even the two closest studies [29,30] failed to meet these criteria because they used different measurement approaches.

#### 2.7.2. Meta-Analysis Approach 

We conducted a meta-analysis to estimate the prevalence of stress and depressive symptoms among undergraduate nursing students. When studies reported only an overall prevalence (without severity categories), we extracted the reported values. When severity-category counts were available, we calculated prevalence by counting the fraction of respondents at or above the predefined cut-offs for “mild” (depressive symptoms), “mild” (stress, measured with DASS-21), and “moderate” (stress, measured with PSS-10). Effect sizes (ES) and 95% confidence intervals (CIs) were calculated using random-effects models [31]. For longitudinal studies that reported prevalence at multiple time points, we extracted only the baseline prevalence estimate per cohort for meta-analysis and reported other time points descriptively. If timepoint-specific prevalence was not reported (e.g., only a complete-case sample across waves), the study was summarized narratively and not pooled for prevalence.

A total of 34 studies were included in the prevalence analysis. Stress prevalence was reported in 22 studies. Of these, 15 were pooled and seven were not pooled due to non-comparable or insufficiently documented instruments and were summarized narratively (Section 3.2). The prevalence of depressive symptoms was reported in 30 studies, all of which were pooled into the meta-analysis.

Subgroup analyses were conducted to explore heterogeneity and variations across studies. These analyses were stratified by symptom severity, study location, assessment instruments, country income groups (as classified by the World Bank), and study quality. Because studies did not consistently report severity distributions using uniform cut-offs (e.g., normal/mild/moderate/severe), and many reported only one or two categories or used different categorical structures, we could not reconstruct mutually exclusive severity categories for meta-analysis. Therefore, each severity category was analyzed independently as the proportion of the total sample meeting that specific cut-off. This approach is commonly used in prevalence meta-analyses when severity reporting is heterogeneous and does not permit a single standardized severity breakdown [3,32].

I^2^ statistics were used to assess heterogeneity, while Begg’s test, Egger’s test, and funnel plots were employed to evaluate publication bias. Due to variability in assessment tools, only studies utilizing psychometrically comparable instruments for depressive symptoms and stress were included in the combined effect size calculation. We defined psychometric comparability as the use of validated instruments with moderate-to-strong convergent validity using the established benchmark measures (approximately r ≈ 0.60–0.80 in validation studies). For perceived stress, convergent validity between the PSS-10 and DASS-21 subscales has been reported (e.g., PSS-10 with DASS-21 stress r ≈ 0.64) [33,34]. For depressive symptoms, acceptable correlations (r > 0.6) among commonly used symptom scales (e.g., PHQ-9, BDI/BDI-II, CES-D, DASS-21, GADS, GHQ-28, HADS, SRDS) have been reported in validation works [26,35,36]. Studies that could not be pooled for meta-analysis were incorporated into the narrative synthesis. All statistical analyses were conducted using STATA 17 [37].

## 3. Results

**Figure 1:**
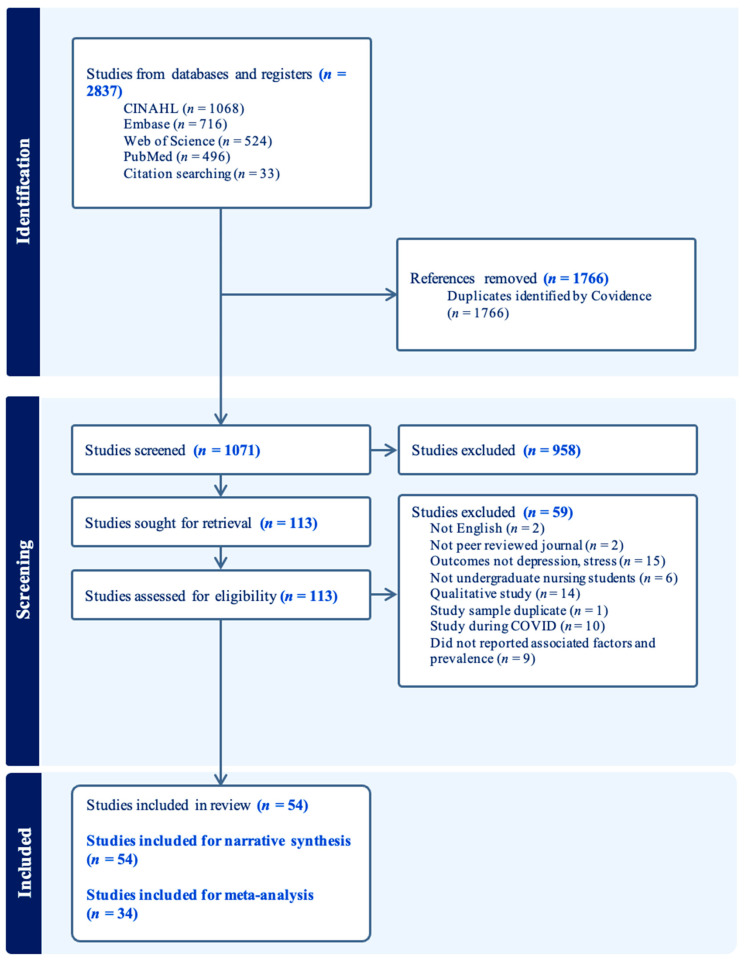
PRISMA flowchart.

After removing duplicates, 1071 records were identified for title and abstract screening. Also, 958 records did not meet the eligibility criteria and were excluded, leaving 113 articles for full-text review. Following further evaluation, 59 articles were excluded due to irrelevant populations and outcomes or the absence of sociodemographic or interpersonal factors or prevalence data. The final selection process resulted in 54 articles being included in this review. Figure 1 depicts the PRISMA selection flowchart.

### 3.1. Study Characteristics

Table 2 presents included study characteristics. Fifty out of 54 studies (92.59%) employed a cross-sectional design, with three utilizing a longitudinal design [4,38,39], and one adopting a mixed-methods approach [5]. In 33 of the 54 studies (61.11%), ≥80% of participants were female; 16 studies (29.63%) included <80% female participants; and 5 studies (9.26%) did not report gender distribution. The mean age range of participants was 18 to 25 years in 39 studies (72.22%). See Supplementary Table S1 for detailed descriptive characteristics for each study.

The geographic distribution of studies varied, with the highest proportion conducted in Asia (33.33%, *n* = 18), followed by Latin America and Europe (16.67%, *n* = 9). Sample sizes ranged from 16 to 1716 participants. Specifically, 4 studies (7.41%) had ≤100 participants, 36 (66.67%) had 101–500 participants, 7 (12.96%) had 501–1000 participants, and 7 (12.96%) had ≥1001 participants. Among the included studies, 33.33% (*n* = 18) assessed depressive symptoms only, 40.74% (*n* = 22) assessed stress only, and 25.93% (*n* = 14) examined both depressive symptoms and stress. The most frequently used assessment instruments were the Depression, Anxiety, and Stress Scale-21 (DASS-21; 20.37%, *n* = 11) and the Perceived Stress Scale-10 (14.81%, *n* = 8). Thirty of 54 included studies (55.56%) were rated as good quality, while 44.44% (*n* = 24) were categorized as having fair quality. No studies were rated as poor quality. Common sources of bias included insufficient sample size justification, lack of participation rate, and failure to adjust statistical analyses for potential confounders (See Supplementary Table S2).

### 3.2. Narrative Synthesis

A total of 54 studies were included in the narrative synthesis, of which 43 examined sociodemographic and interpersonal factors associated with stress and depressive symptoms (summarized in Table 3). In addition, seven studies were excluded from pooling due to the use of non-standardized stress instruments that either (1) lacked English-language psychometric references, (2) did not report psychometric properties, or (3) were not validated against commonly used stress scales such as the DASS-21 or PSS-10. These studies were retained for the narrative synthesis. Their reported stress prevalence ranged from 58.4% to 100% [40,41,42,43,44,45,46]. For longitudinal studies, Sonmez et al. [39] reported depressive symptom prevalence at all time points. The baseline prevalence was included in the meta-analysis, and the prevalence of depressive symptoms at follow-up was reported descriptively (19.4%). Dong et al. [38] did not report prevalence at any time points and summarized them narratively. Olvera Alvarez et al. [4] reported baseline prevalence only and was included in the meta-analysis.

#### 3.2.1. Sociodemographic Factors with Depressive Symptoms or Stress

Demographic Factors. Evidence on demographic factors (age, gender, ethnicity, marital status, residence location, and year of study) was mixed. See Table 3 for details.

*Age.* Across 30 outcome-specific analyses (stress: 16; depressive symptoms: 14), 18 out of 30 reported no statistically significant associations. Among significant findings (*n* = 12), the most frequently reported associations were lower depressive symptoms [5,30,47] and lower stress [5,48] among older students (*n* = 5 out of 12).

*Gender.* Across 35 outcome-specific analyses (stress: 19; depressive symptoms: 16), 22 reported no statistically significant associations. Among significant findings (*n* = 13), female students reported higher depressive symptoms [4,29,47,49] and stress levels [14,40,46,47,50,51,52,53] than male students (*n* = 12 out of 13).

*Ethnicity.* Across 8 outcome-specific analyses (stress: 2; depressive symptoms: 6), 7 reported no statistically significant associations. The single significant analysis reported higher depressive symptoms among students from minority ethnic groups compared to White students [5].

*Marital Status*. Across 20 outcome-specific analyses (stress 10; depressive symptoms 10), 15 reported no statistically significant associations. Among significant findings (*n* = 5), the most frequently reported associations were higher depressive symptoms [15,54] and greater stress levels [51] among single students compared to married or partnered students (*n* = 3 out of 5).

*Residence location*. Across 11 outcome-specific analyses (stress 5; depressive symptoms 6), 9 reported no statistical associations. The remaining 2 out of 11 significant findings are from a single study that assessed both depressive symptoms and stress, reporting higher depressive symptoms and greater stress levels among students living in metropolitan areas [15].

*Year of study*. Across 20 outcome-specific analyses (stress 10; depressive symptoms 10), 8 reported no statistical associations, and 12 out of 20 reported significant findings. Among these (*n* = 12), four patterns were reported: (1) Year 1 students had higher depressive symptoms [47] and/or stress levels [47,55] (*n* = 3 out of 12; including [47] that assessed both outcomes); (2) Year 2 students had higher depressive symptoms [6,54,56] and/or stress levels [40] (*n* = 4 out of 12); (3) students in 3rd, 4th, or 5th year experienced elevated stress levels [46,53,57] (*n* = 3 out of 12); (4) two studies reported significant associations between semester with stress level [41,58].

Financial Factors. Evidence on financial factors (income, financial issues, and working) yielded mixed results.

*Income and Financial Issues.* Across 19 outcome-specific analyses (stress 9; depressive symptoms 10), 8 reported no statistically significant associations. Among significant findings (*n* = 11), lower income and financial challenges were associated with higher depressive symptoms [15,47,58,59,60] and stress levels [46,47,55,58] (*n* = 10 out of 11).

*Working.* Across 15 outcome-specific analyses (stress 7; depressive symptom 8), 12 reported no statistically significant associations. Among significant findings (*n* = 3), the most frequently reported associations were higher depressive symptoms [56] and stress [55] among students who were employed (*n* = 2 out of 3).

**Table 3:** Summary of sociodemographic and interpersonal factors associated with stress levels and depressive symptoms.

	Stress			Depressive Symptoms		
Factor	Results (*n*)	Significant Results	Non-Significant Results	Results (*n*)	Significant Results	Non-Significant Results
Demographic Factors						
Age	16	(older) ↓ (stress): [5,48] (older) ↑ (stress): [61] (younger) ↑ (stress): [14,15]	[18,38,46,47,51,52,53,55,60,62,63]	14	(older) ↓ (DS): [5,30,47] (older) ↑ (DS): [63,64] (younger) ↑ (DS): [15] (age) ? DS: [56]	[18,54,60,65,66,67,68]
Gender	19	(female) ↑ (stress): [14,40,46,47,50,51,52,53]	[15,38,42,48,55,60,61,63,69,70,71]	16	(female) ↑ (DS): [4,29,47,49] (male) ↑ (DS): [54]	[15,30,56,59,60,63,65,66,67,68,72]
Ethnicity	2	NR	[15,46]	6	(non-White) ↑ (DS): [5]	[4,15,29,30,68]
Marital Status	10	(single) ↑ (stress): [51] (partnered) ↑ (stress): [73] (status) ? (stress): [14]	[42,46,47,48,53,55,63]	10	(single) ↑ (DS): [15,54]	[30,47,63,64,65,66,67,68]
Residence Location	5	(metropolitan) ↑ (stress): [15]	[18,38,47,70]	6	(metropolitan) ↑ (DS): [15]	[18,47,56,59,66]
Year of study	10	(year 2) ↑ (stress): [40] (year 1) ↑ (stress): [47,55] (3rd, 4th, or 5th year) ↑ (stress): [46,53,57] (year) ? (stress): [41,58]	[60,70]	10	(year 2) ↑ (DS): [6,54,56] (year 1) ↑ (DS): [47]	[29,57,58,60,65,74]
Financial Factors						
Income	3	(lower income/monthly income) ↑ (stress): [46,55]	[70]	5	(lower income) ↑ (DS): [15,59] (higher income) ↑ (DS): [68]	[30,67]
Financial issues	6	(financial crisis/income less than expense) ↑ (stress): [46,47,58]	[18,60,62]	5	(financial crisis/income less than expense/poor economic situation) ↑ (DS): [47,58,60]	[4,18]
Working	7	(working) ↑ (stress): [55]	[42,46,51,52,60,63]	8	(part-time) ↑ (DS): [56] (work hours) ? (DS): [49]	[4,15,60,63,64,68]
Family Situation Factors						
Number of children	2	NR	[42,63]	3	(no children) ↑ (DS): [15]	[63,66]
Living arrangement	2	NR	[46,52]	3	(living with parent/spouse) ↓ (DS): [15,68]	[30]
Housing security	0	–	–	1	(housing stressors) ? (DS): [4]	NR
Type of family	2	NR	[18,47]	1	NR	[18]
Parental education level	2	(higher parental level) ↓ (stress): [38]	[47]	1	NR	[59]
Health Factors						
Health status	5	(chronic illness) ↑ (stress): [15] (poor perceived health status) ↑ (stress): [60,71] (poor perceived mental health) ↑ (stress): [60]	[18]	6	(received treatment) ↑ (DS): [4] (chronic illness) ↑ (DS): [15] (hospitalized) ↑ (DS): [59] (poor perceived health status) ↑ (DS): [60] (poor perceived mental health) ↑ (DS): [60]	[18]
Family mental illness	1	NR	[18]	2	NR	[18,59]
Psychotropic drug	0	NR	NR	3	(psychotropic drugs) ↑ (DS): [29,30,66]	NR
Interpersonal Factors						
Family relationship	3	(support) ↓ (stress): [73] (relationship) ↓ (stress): [75]	[18]	3	(relationship) ↓ (DS): [75]	[18,59]
Friendship	3	(support) ↓ (stress): [75] (conflicts) ↑ (stress): [18]	[71]	3	(conflicts) ↑ (DS): [18] (less friends) ↑ (DS): [71]	[75]
Social activities	–	–	–	2	(dissatisfied with social activities) ↑ (DS): [39]	[65]
Romantic relationship	2	(Break up) ↑ (stress): [18]	[60]	3	(Break up or relationship issues in past year) ↑ (DS): [59]	[18,60]

Note: See Supplementary Tables S3 and S4 for detailed results; Numbers in brackets refer to cited articles. Symbols refer to cases of a factor associated with increased symptom levels (↑), a factor associated with reduced symptom levels (↓), and a significant association with unclear directionality (?).

Family Situation Factors. Evidence on family situations (number of children, living arrangements, housing security, family type, parental education level) yielded mixed findings. Across 17 outcome-specific analyses (stress 8; depressive symptoms 9), 12 reported no statistically significant associations. The remaining 5 out of 17 significant findings were isolated and included: lower stress with higher parental education level [38]; higher depressive symptoms among students with no children [15]; lower depressive symptoms among students living with a parent/spouse [15,68]; and higher depressive symptoms associated with housing insecurity [4].

Health Factors. Evidence on health factors (health status, psychotropic drug use, and family mental illness history) was largely consistent. Across 17 outcome-specific analyses (stress 6; depressive symptom 11), 5 reported no statistically significant associations. Baruah et al. [18] accounted for 4 out of 5 null analyses because they examined health status and family mental illness in relation to both stress and depressive symptoms. All the rest of the studies reported that having health issues (chronic illnesses, poor mental health/physical health, using psychotropic drugs) was associated with higher depressive symptoms [4,15,29,30,59,60,66] and/or higher stress levels [15,60,71] (*n* = 12 out of 17; study [60] assessed both physical and mental health and was counted twice).

#### 3.2.2. Interpersonal Factors with Depressive Symptoms or Stress

Across 19 outcome-specific analyses examining interpersonal factors, 9 reported no statistically significant associations. Among significant findings (*n* = 10 out of 19), family-related factors (e.g., greater support/better relationships) [73,75] and friendship-related (numbers of friends, friend supports, social activities) factors [18,39,71,75] were most frequently associated with lower depressive symptoms or stress (*n* = 6 out of 10). Two analyses reported that relationship breakup was associated with higher depressive symptoms [59] or stress levels [18].

### 3.3. Meta-Analysis

#### 3.3.1. Prevalence of Depressive Symptoms and Stress

**Figure 2:**
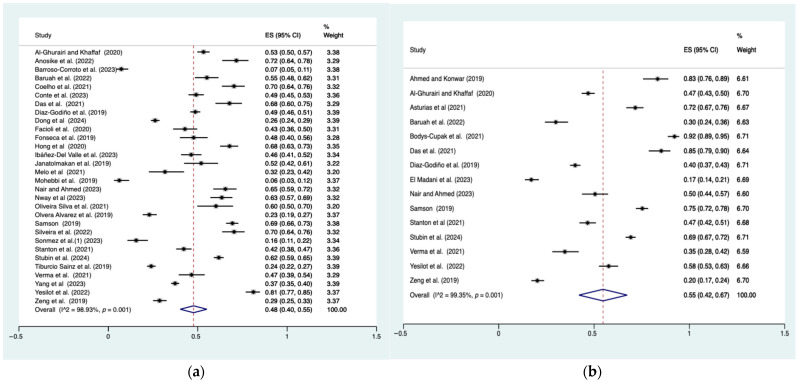
Prevalence of depressive symptoms and stress. (**a**) Prevalence of depressive symptoms (**b**) Prevalence of stress. Note: Pooled prevalence included 34 studies: [4,5,6,14,15,18,29,30,39,47,49,54,55,57,58,59,60,62,63,64,66,67,68,72,74,75,76,77,78,79,80,81,82,83]. Dots are the effect size (ES) for each study, with horizontal lines indicating the 95% CI. Diamonds at the bottom are the pooled effect size, with their width indicating the 95% CI. Vertical dashed line marks are the overall pooled estimate.

For depressive symptoms, 30 studies were included in the effect size calculation, with a sample size ranging from 88 to 1716, totaling 13606 participants. For stress, 15 studies were included, with sample sizes ranging from 120 to 1193, totaling 7195 participants. The prevalence estimates for depressive symptoms and stress were presented in Figure 2. All I^2^ statistics exceeded 80%, indicating high heterogeneity across studies. The prevalence of depressive symptoms was 48% (95% CI: 40–55%; I^2^ = 98.93%, *p* < 0.01), while the prevalence of stress was 55% (95% CI: 42–67%; I^2^ = 99.35%, *p* < 0.01).

#### 3.3.2. Prevalence of Depressive Symptoms and Stress: Subgroup Analyses

Table 4 presents subgroup analyses of depressive symptoms and stress. Among all students, category-specific prevalence estimates for depressive symptoms (based on instrument-defined severity cut-offs) were 22% for mild symptoms (95% CI: 18–25%; I^2^ = 93.58%, *p* < 0.01), 23% for moderate symptoms (95% CI: 19–26%; I^2^ = 90.66%, *p* < 0.01), and 16% for severe symptoms (95% CI: 11–20%; I^2^ = 97.24%, *p* < 0.01) (Supplementary Figure S1). Furthermore, subgroup analyses were performed to explore sources of heterogeneity related to study location, country income level, assessment tools, and study quality. Pooled prevalence was higher in lower–middle-income countries (63%, 95% CI: 57–69%), and studies using the Beck’s Depression Inventory-II (BDI-II) (68%, 95% CI: 63–74%). Estimates were similar across study quality (Fair 47% vs. Good 48%) (Supplementary Figure S2).

Prevalence varied by severity level for stress. Among all students, category-specific prevalence estimates for stress levels (based on instrument-defined severity cut-offs) were 16% for mild stress (95% CI: 13–19%; I^2^ = 89.96%, *p* = 0.01), 22% for moderate stress (95% CI: 17–26%; I^2^ = 95.17%, *p* < 0.01), and 18% for severe stress (95% CI: 11–26%; I^2^ = 99.03%, *p* < 0.01) (Supplementary Figure S3). Subgroup analyses examined variation by study location, country income level, assessment tools, and study quality. Pooled prevalence was higher in high-income countries (70%, 95% CI: 52–88%), and in studies using the PSS-10 assessment tool (66%, 95% CI: 28–104%) compared with DASS-21. Estimates were similar across study quality (Fair 56% vs. Good 53%) (Supplementary Figure S4).

#### 3.3.3. Publication Bias

Regarding the funnel plot (Supplementary Figure S5), there was some symmetry, suggesting minimal publication bias. Results from statistical tests further supported this finding. Begg’s test showed *p* = 0.392 for depressive symptoms and *p* = 0.767 for stress, while Egger’s test yielded *p* = 0.078 for depressive symptoms and *p* = 0.813 for stress. These findings suggest no significant evidence of publication bias.

## 4. Discussion

This systematic review and meta-analysis found that undergraduate nursing students reported a high prevalence of depressive symptoms and stress. Sociodemographic and interpersonal factors associated with these outcomes were mixed. Reported significant associations included younger age, female gender, single status, financial strain, poor health, limited social support, relationship conflict, and dissatisfaction with social activities. Subgroup analyses revealed that depressive symptoms were more prevalent in studies conducted in lower-income countries and those using the BDI-II. In contrast, higher stress levels were observed in high-income countries and in studies utilizing the PSS-10.

It is also important to note that these findings should be interpreted in light of the methodological quality of the included studies. Common risk-of-bias concerns (e.g., lack of sample size justification, lack of reported participation rate) may affect the accuracy and representativeness of prevalence estimates and may contribute to heterogeneity. In addition to these methodological issues, residual variability likely reflects unmeasured contextual differences across studies, including cultural norms around emotional disclosure, variation in academic and clinical training structures, institutional support, and broader socioeconomic or political conditions.

### 4.1. Sociodemographic Factors

Demographic Factors. Overall, evidence for demographic associations of depressive symptoms and stress was mixed across included studies, with many reporting null associations and a subset reporting significant effects. Younger age, female gender, and single status were among the factors reported to be associated with increased depressive symptoms and higher stress levels. These findings align with previous research showing that younger female nursing students often experience elevated stress levels [84]. Additional studies on undergraduate students have also demonstrated that younger females reported higher levels of depression [85,86].

Most undergraduate nursing students are under 25 years old, a developmental stage characterized by the transition from adolescence to adulthood [23,85]. During this period, students take on increasing responsibility for their career goals, academic workloads, and future employment, which may increase stress levels [85,87]. Single status has also been identified as a factor associated with increased depressive symptoms and stress, likely due to feelings of loneliness and reduced social support [88]. Younger female undergraduate nursing students may be more vulnerable to stress and depressive symptoms due to less developed coping mechanisms, the challenges of living away from home for the first time, and reliance on social support from friends that may be less stable than family support [89].

In addition, associations between year of study and stress and depressive symptoms were inconsistent, with significant findings distributed across early (Year 1–2) and later (Year 3–5) years. These mixed findings likely reflect the evolving nature of stressors encountered throughout nursing education. First- and second-year students are adjusting to a new academic environment. They may experience transition-related stress as they enter nursing education, including the early introduction of clinical rotations in countries such as the United States and Canada [90,91]. In contrast, in countries like China, clinical rotations typically occur in the final year of study [60], which may shift the timing and type of stressors students experience. Additionally, students in later years also face academic pressures associated with graduation requirements, securing employment, and the successful completion of nursing licensure examinations [90,91,92].

Financial Factors. Lower income and financial challenges were consistent factors identified to be associated with higher stress and depressive symptoms. Additionally, studies examining income and financial difficulties were conducted in India, Australia, China, Turkey, and Brazil, suggesting that financial strain is a concern for nursing students across diverse economic and cultural contexts. These findings align with prior research [85,93], which identified a strong association between financial hardship and heightened psychological distress among global college students. Nursing students often face financial challenges, including expenses related to tuition, course materials, food, housing, and transportation to clinical sites [46]. The cumulative strain of these economic pressures may impair students’ academic performance and mental well-being. This underscores the need for institutional support systems to provide financial assistance, mental health resources, and stress management strategies.

Family Situation Factors. Overall, family situation factors were infrequently associated with outcomes. Significant findings suggested higher stress and/or depressive symptoms among students without children and those with housing insecurity. In contrast, living with a parent/spouse was linked to lower depressive symptoms. Prior research demonstrates that living arrangements and housing security [85] may influence mental health outcomes among undergraduate students. Students who reside with their parents or partners tend to benefit from stronger emotional support and financial stability, which may help protect them against psychological distress [85]. By comparison, undergraduate students often move out for the first time. They may live with roommates or friends, a relationship that can be less stable than family and may increase their risk of psychological distress [89].

Health Related Factors. Poor perceived physical or mental health was consistently identified as being associated with stress and depressive symptoms in our review. These results align with the existing literature, which highlights poor physical health as a significant contributor to psychological distress among undergraduate students [85]. These findings emphasize the need for comprehensive mental health interventions, targeted wellness programs, and institutional support to address physical and psychological well-being within nursing education.

### 4.2. Interpersonal Factors

Although research on the relationship between interpersonal factors and depressive symptoms or stress among undergraduate nursing students only included eight studies, relationships with family and friends had significant associations with stress and depressive symptoms. These results align with a previous systematic review, which demonstrated that a lack of supportive social networks and reduced engagement in social activities significantly contribute to depressive symptoms and stress among undergraduate students [85]. Similarly, Wang et al. [94] identified social support from family, friends, and significant others as a key determinant of mental health in this population. These findings suggest that incorporating peer-support initiatives into nursing curricula or student services (e.g., peer mentoring and facilitated support groups) could be a feasible strategy to promote well-being.

### 4.3. Prevalence Results of Depressive Symptoms and Stress

Our review indicates that depressive symptoms were common among undergraduate nursing students (pooled prevalence 48%). Among all students, 23% were classified as having moderate depressive symptoms and 16% as having severe symptoms. This prevalence is higher than that reported by Tung et al. [21], who found that 34% of nursing students exhibited depressive symptoms. The difference may be because Tung et al. [21] did not limit their search by publication date and included nursing students enrolled in various educational programs (e.g., diploma, associate degree, and graduate students).

Additionally, the higher prevalence of depressive symptoms observed in our study may reflect increased mental health awareness and reduced stigma in contemporary student populations, potentially resulting in greater self-reporting of psychological distress among nursing students [95]. Further, the evolving demands of healthcare education, including high-stakes examinations and the integration of advanced clinical technologies, may intensify depressive symptoms among nursing students [96]. Finally, although COVID-19-related studies were excluded, the long-term impact of the pandemic—including social isolation, disrupted learning environments, and heightened uncertainty regarding future healthcare roles—may have indirectly contributed to increased psychological distress among nursing students, even in studies conducted outside the pandemic period [97].

Our review reports that stress was common among undergraduate nursing students (pooled prevalence 55%). In the overall student sample, 22% were classified as having moderate stress levels and 18% as having severe stress levels. These results differ from those of Vo et al. [3], who found that 42.1% of nursing students were classified as having moderate stress and 19.5% as having severe stress. Several factors may explain these differences. The search strategy in Vo et al. [3] was not limited by publication date, included studies published in four languages (i.e., English, Chinese, Vietnamese, and Korean), and included all types of nursing students (e.g., diploma, associate degree, and graduate students), capturing a wider range of regional and cultural contexts. In contrast, our study focused specifically on undergraduate nursing students from diverse international settings, providing a more targeted understanding of stress and depressive symptoms in this population. Although our stress rates are lower than reported in Vo et al. [3], they still indicate that a substantial proportion of undergraduate nursing students experience moderate stress levels. This highlights ongoing mental health challenges in this population and underscores the need for our study to examine current prevalence and correlates of stress among undergraduate nursing students.

Subgroup analysis in this review was guided by a prior systematic review on mental health outcomes among undergraduate students [98], highlighting the importance of stratifying outcomes by country income level, geographic region, assessment tools, and study quality. Our analysis found that higher levels of depressive symptoms were reported in studies conducted in lower–middle-income countries and those using the BDI-II assessment tool. In contrast, higher stress levels were observed in studies from high-income countries and those utilizing the PSS-10. These patterns may reflect a combination of contextual and methodological factors—particularly those related to income level and instrument, which are discussed below.

Financial and structural challenges may drive the elevated prevalence of depressive symptoms in lower–middle-income countries. Students in these settings may experience greater financial hardship due to lower family incomes and limited access to scholarships or financial aid opportunities [98]. Additionally, reduced availability of mental health services and early intervention options [99] may also contribute to sustained psychological distress in these regions. Notably, in our results, while stress levels in this group were not the highest overall, they remained substantial (54%), which may help explain the elevated depressive symptom burden.

In contrast, the higher stress levels in high-income countries may reflect their acute academic and clinical demands. Nursing students in these contexts often encounter rigorous curricula, early clinical exposure, and heightened expectations for performance and adaptability [96]. However, greater access to mental health resources and social support systems in these countries may buffer the progression from stress to depressive symptoms, which could account for the relatively lower prevalence of depressive symptoms despite high stress levels [100].

Methodological differences in measurement tools may also account for variation in reported prevalence. Studies using the BDI-II reported the highest prevalence (68%), likely due to its development for clinical populations and its inclusion of a wide range of affective and cognitive symptoms, making it more sensitive than general tools like the PHQ-9 [101] or GHQ-28 [35].

For stress, studies using the PSS-10 reported a higher pooled prevalence (66%) than those using the DASS-21 (51%). This may reflect the PSS-10’s conceptualization of stress as a broader, subjective appraisal of unpredictability and overload over the past month [102], compared to the DASS-21, which focuses on specific symptoms like irritability and tension over the past week [103]. As a result, PSS-10 may capture broader, milder, and situational stress experiences, leading to higher prevalence estimates. In support of this, one study found a moderate correlation between PSS-10 and DASS-21 stress subscales (r = 0.64). It was also noted that PSS-10 further correlates with DASS-21 depression (r = 0.61) and anxiety (r = 0.54) subscales, indicating that PSS-10 reflects a broader emotional response than DASS-21 [34].

### 4.4. Gaps in Research and Future Considerations

The included studies highlight several knowledge gaps that warrant further investigation. First, the predominance of cross-sectional study designs limits the ability to assess longitudinal changes in stress responses and depressive symptoms among nursing students. Future research should incorporate longitudinal approaches to capture short-term variations (e.g., within semester, or around midterm and final exams) and long-term trends across nursing programs. In addition, many included studies in our review did not specify when data were collected during the academic term. Reporting the timing of data collection will be essential for understanding patterns of stress and depressive symptoms among nursing students. Second, all included studies relied on self-reported questionnaires for assessing mental health symptoms, rather than the gold standard of clinician-administered diagnostic interviews [104]. Future research should consider incorporating structured clinical interviews when feasible and using validated brief screening tools and online assessment modules, such as digital versions of standardized screening tools, to increase the accuracy of mental health assessments in this population. Additionally, findings revealed variability in the prevalence of depressive symptoms and stress depending on the self-reported assessment tools used. This inconsistency highlights the need for research to determine the most effective and reliable screening instruments for evaluating mental health symptoms among nursing students. Widely used and well-validated instruments such as the PSS-10, PHQ-9, and DASS-21 may be preferred because they are commonly used across both student and general populations, thus facilitating comparisons across studies [105,106,107]. From an implementation standpoint, the PHQ-9 is widely used in primary care clinics [107] and could be considered for incorporation into student health services to support standardized screening and follow-up [108].

Despite the established role of sociodemographic factors in nursing students’ stress and depressive symptoms, research remains limited. Only one study [4] identified an association between housing security and depressive symptoms, while no studies have examined the impact of housing and food security on stress levels and depressive symptoms in this population. Existing research on undergraduate healthcare and medical students, who are also navigating rigorous academic and clinical demands, has demonstrated that food and housing insecurity significantly contribute to psychological distress [109,110]. Furthermore, these factors are recognized as key social determinants of mental health [111]. Future research should assess baseline food and housing security among nursing students and their associations with mental health outcomes, providing evidence for student-focused interventions that address these critical determinants of well-being.

Our findings suggest that interpersonal factors such as friendships and family relationships protect against elevated stress and depressive symptoms among nursing students. However, critical gaps remain in understanding the influence of discrimination and social loneliness on mental health in this population. Qualitative studies suggest that nursing students frequently experience perceived discrimination [17] and social loneliness [112]. Despite these findings, no quantitative research has examined the relationships between these factors and stress or depressive symptoms among undergraduate nursing students. Future studies should adopt a holistic approach incorporating qualitative and quantitative methods to assess the broader interpersonal and social determinants of mental health in nursing education.

### 4.5. Study Implications

Our findings indicate that undergraduate nursing students experience high levels of stress and depressive symptoms, with multiple sociodemographic factors contributing to these mental health challenges. When compared with global college students, nursing students appear to be more burdened: a recent meta-analysis reported a higher prevalence of depressive symptoms of 48% vs. 33.6% [98] and stress of 55% vs. 36.34% [113].

It is thus important to address nursing students’ stress and depressive symptoms through targeted interventions—such as stress-management training, coping-skills programs, or digital mental health tools—which can help reduce stress and improve mental well-being in this population. Emerging work has begun to evaluate such individual-level interventions [114], but further studies are needed to examine their effectiveness, feasibility, and acceptability across diverse nursing programs and contexts to ensure they meaningfully reduce stress and depressive symptoms among nursing students.

Furthermore, educational institutions should implement comprehensive, system-level mental health programs tailored to the unique needs of nursing students. For example, they can develop supportive policies that address academic workload and scheduling challenges. Nursing programs should incorporate regular health screenings that assess both depression and stress. While depression is routinely screened in clinical settings, stress is often overlooked despite its strong association with academic burnout and emotional exhaustion [20]. Including stress in mental health assessments within nursing programs can facilitate earlier identification of students at risk and enable timely, targeted interventions. In addition, structured peer mentorship programs and faculty training should be established to recognize and respond to student distress. By fostering a supportive environment, institutions can help mitigate stress and depressive symptoms, ultimately enhancing academic performance and overall quality of life for future healthcare professionals.

### 4.6. Study Limitations

This systematic review has several limitations. First, substantial heterogeneity was observed in our prevalence meta-analysis, likely reflecting differences in study design, sample characteristics, cultural and geographic factors, assessment tools, and study periods. Variability in study quality, including unclear response rates and inconsistent analytic adjustments, may further obscure true prevalence estimates. In addition, because prevalence was sometimes extracted directly from studies and sometimes derived using harmonized instrument-specific severity cut-offs, pooled estimates may be over- or under-estimated and should be interpreted with caution. Second, while our inclusion criteria focused on undergraduate nursing students, some studies did not explicitly use the term “undergraduate.” In these cases, enrollment in entry-level nursing programs or age distributions implied undergraduate status. Third, for many sociodemographic and interpersonal factors, only a small number of studies with modest sample sizes were available, limiting statistical power and cautioning against over-interpretation of both significant and non-significant findings. Fourth, observed associations (e.g., female gender, single status, having no children) may also reflect the demographic homogeneity of nursing student populations rather than independent risk effects. Fifth, this review did not synthesize all potentially relevant factors. Program-level training characteristics (e.g., curriculum intensity, course load, timing of clinical exposure) and program types (e.g., accelerated vs. traditional; program length) were rarely reported in a standardized or extractable format, limiting opportunities for pooled analysis. In addition, unmeasured contextual factors, such as differences in curriculum design, variation in the timing and intensity of clinical training, institutional expectations for early clinical exposure, and broader educational system structures, likely contribute to the inconsistent reporting of these program-level variables across studies. Because of this heterogeneity, these factors could not be incorporated into either the meta-analytic models or the narrative synthesis, and their absence reflects limitations in the underlying evidence base rather than an omission in our analytic approach. Sixth, co-occurring stress and depressive symptoms, and substance use were also not synthesized because most studies did not report joint prevalence or cross-tabulated data. Seventh, the geographic distribution of included studies was skewed toward middle- and upper–middle-income countries, limiting the global generalizability of our pooled estimates to low-income settings. Eighth, the review was restricted to English-language publications and to studies published after 2019, which may have led to the omission of earlier high-quality research. Lastly, the decision not to include PsycINFO, due to overlap with other databases and the nursing-specific coverage provided by CINAHL, may also have excluded some psychology-focused studies relevant to stress and depressive symptoms.

## 5. Conclusions

Our study underscores the significant prevalence of stress and depressive symptoms among nursing students, reinforcing the need for faculty-led stress management inter-vention programs. Nearly half of undergraduate nursing students worldwide experience stress and depressive symptoms. This systematic review revealed variations in prevalence by region, country income, and measurement tools, extending prior evidence on global disparities in these outcomes. Financial hardship, poor perceived health, and limited social connectedness emerged as key modifiable factors. Future research should focus on implementing culturally tailored interventions that strengthen support systems and promote students’ mental well-being.

## Figures and Tables

**Table 1:** Search strategy.

Database	Search Terms
PubMed	(“students, nursing” [Mesh] OR “nursing student *”) AND (“anxiety” OR “anxiety” [MeSH Terms] OR “depression” [MeSH Terms] OR “depression” OR “depressive” OR stress OR “Stress, Psychological” [Mesh]) AND (“Sociodemographic Factors” [Mesh] OR “Sociological Factors” [Mesh] OR “sociological factors” OR “Socioeconomic Factors” [Mesh] OR “sociodemographic” OR “socioeconomic” OR “poverty” OR “Poverty” [Mesh] OR “food security” OR “Food Security” [Mesh] OR “food insecurity” OR “Food Insecurity” [Mesh] OR “housing” OR “Housing” [Mesh] OR “Ethnic and Racial Minorities” [Mesh] OR “minority” OR “minorities” OR “discrimination” OR “Social Discrimination” [Mesh] OR “racism” OR “loneliness” OR “Loneliness” [Mesh] OR “social isolation” OR “Social Determinants of Health” [Mesh] OR “social determinants” OR “Family Relations” [Mesh] OR “family relations” OR “Friends” [Mesh] OR “friends”) NOT covid
Embase	(‘nursing student’/exp OR ‘nursing student’) AND (‘anxiety’/exp OR ‘anxiety’ OR ‘depression’/exp OR depression OR ‘depressive’ OR ‘stress’/exp OR stress OR ‘mental stress’/exp) AND (‘sociodemographics’/exp OR ‘sociodemographics’ OR ‘socioeconomics’/exp OR ‘socioeconomics’ OR ‘social aspects and related phenomena’/exp OR ‘social aspects and related phenomena’ OR ‘sociological factors’ OR ‘poverty’/exp OR ‘poverty’ OR ‘food security’/exp OR ‘food security’ OR ‘food insecurity’/exp OR ‘food insecurity’ OR ‘housing’/exp OR ‘housing’ OR ‘ethnic’ OR ‘ancestry group’/exp OR ‘minority’ OR ‘minorities’ OR ‘discrimination’/exp OR ‘discrimination’ OR ‘social discrimination’/exp OR ‘racism’/exp OR ‘racism’ OR ‘loneliness’/exp OR loneliness OR ‘social isolation’/exp OR ‘social isolation’ OR ‘social determinants of health’/exp OR ‘social determinants’ OR ‘family relation’/exp OR ‘family relations’ OR ‘friend’/exp OR ‘friends’) NOT (‘coronavirus disease 2019’/exp OR ‘coronavirus disease 2019’ OR covid) AND (‘article’/it OR ‘article in press’/it)
Web of Science	(“nursing student *”) AND (“anxiety” OR “depression” OR “depressive” OR stress) AND (“sociological factors” OR “sociodemographic” OR “socioeconomic” OR “poverty” OR “food security” OR “food insecurity” OR “housing” OR “minority” OR “minorities” OR “discrimination” OR “racism” OR “loneliness” OR “social isolation” OR “social determinants” OR “family relations” OR “friends”) NOT covid
CINAHL	(“nursing student *” OR MH “Students, Nursing+”) AND (anxiety OR MH “Anxiety+” OR depression OR MH “Depression+” OR depressive OR stress OR MH “Stress, Psychological+”) AND (sociodemographic OR MH “Sociodemographic Factors” OR socioeconomic OR MH “Socioeconomic Factors+” OR “sociological factors” OR poverty OR MH “Poverty+” OR “food security” MH “Food Security+” OR “food insecurity” OR housing OR MH “Housing Instability” OR minority OR minorities OR MH “Minority Groups+” OR MH “Discrimination” OR discrimination OR racism MH “Racism+” OR loneliness OR MH “Loneliness” OR “social isolation” OR MH “Social Isolation+” OR “social determinants” OR MH “Social Determinants of Health” OR “family relations” OR MH “Family Relations+” OR friends) NOT covid

**Table 2:** Characteristics of included studies.

	No. Reports (*n*)	Percentage (%)
Total number of studies	54	100
Design (*n* = 54)		
Cross-sectional study	50	92.59
Longitudinal study	3	5.56
Mix-method study	1	1.85
Gender (*n* = 54)		
≥80% Female	33	61.11
<80% Female	16	29.63
Not Reported	5	9.26
Age (*n* = 54)		
Age mean (18–25)	39	72.22
Age % (Age reported categorical % only)	9	16.67
Not Reported	6	11.11
Age % (Age reported categorical % and mean/sd) (*n* = 11)		
≥50% participants in early adult age band (18–25)	9	81.82
<50% participants in early adult age band (18–25)	2	18.18
Region (*n* = 54)		
Africa (Morocco, Nigeria)	3	5.56
Asia (Hong Kong, India, China Mainland, Myanmar, South Korea, and China Taiwan)	18	33.33
Australasia (Australia)	2	3.70
Europe (Greece, Italy, Poland, and Turkey)	9	16.67
Latin America (Brazil, Peru)	9	16.67
Middle East (Iran, Iraq, Israel, Jordan, and Saudi Arabia)	8	14.81
North America (Mexico and United States)	5	9.26
Sample size (*n* = 54)		
≤100	4	7.41
101–500	36	66.67
501–1000	7	12.96
≥1001	7	12.96
Assessed mental health (*n* = 54)		
DS Only	18	33.33
Stress Only	22	40.74
DS & Stress	14	25.93
Assessment instrument (*n* = 57) ^a^		
CES-D	5	9.26
DASS-21	11	20.37
PHQ-9	3	5.56
BDI	4	7.41
BDI-II	3	5.56
PSS-10	8	14.81
PSS-14	2	3.70
Other DS instruments ^b^	6	11.11
Other Stress instruments ^c^	15	27.78
Study overall risk of bias (*n* = 54)		
Fair	24	44.44
Good	30	55.56

Note: ^a^ Percentages are based on 54 unique studies. Three studies used two instruments; therefore, totals exceed 54; ^b^ GADS, Goldberg Anxiety and Depression Scale; GHQ-28, General Health Questionnaire-28; HADS, Hospital Anxiety & Depression Scale; SRDS, Self-Rated Depression Scale. ^c^ ASIS, Academic stress inventory scale; ASNS, Assessment of Stress in Nursing Students; ASUSQ, About Stress in University Students questionnaire; C-PSQ, Chinese version of the Perceived Stress Questionnaire; DSI, Daily Stress Inventory; NUSS, Nursing University Stress Scale; PSS-Sheu et al., Perceived Stress Scale for Nursing Students, SINS-CN, Stressors in Nursing Students Scale-Chinese version; SNSI, Student Nurse Stress Index Scale; SSKNS, Stress Scale for Korean Nursing Student; TC-SINS, Traditional Chinese Version-Stressors in Nursing Students; Abbreviation = BDI, Beck’s Depression Inventory; BDI-II, Beck’s Depression Inventory-II; CES-D, Center for Epidemiologic Studies Depression Scale; DASS-21, Depression Anxiety Stress Scale 21; PHQ-9, Patient Health Questionnaire-9; PSS-10, Perceived Stress Scale 10; PSS-14, Perceived Stress Scale 14; DS, Depressive Symptoms.

**Table 4:** Subgroup analyses of the prevalence of depressive symptoms and stress levels among undergraduate nursing students.

Subgroup Comparison	Pooled Prevalence of Depressive Symptoms				Pooled Prevalence of Stress Levels			
	# of Studies	Pooled Prevalence	95% CI		# of Studies	Pooled Prevalence	95% CI	
			Lower Limit	Upper Limit			Lower Limit	Upper Limit
Severity level								
Mild DS/Stress	18	0.22	0.18	0.25	11	0.16	0.13	0.19
Moderate DS/Stress	16	0.23	0.19	0.26	12	0.22	0.17	0.26
Severe DS/Stress	17	0.16	0.11	0.20	12	0.18	0.11	0.26
Study location								
Asia	10	0.53	0.41	0.64	7	0.54	0.32	0.76
Australasia	1	0.42	0.38	0.47	2	0.59	0.55	0.62
Europe	5	0.40	0.11	0.69	2	0.83	0.81	0.86
Latin America	7	0.53	0.44	0.63	1	0.40	0.37	0.43
North America	3	0.36	0.10	0.62	1	0.69	0.67	0.72
Middle East	3	0.37	0.02	0.72	1	0.47	0.43	0.50
Africa	1	0.72	0.64	0.78	1	0.17	0.14	0.21
Country income								
High	7	0.42	0.25	0.60	4	0.70	0.52	0.88
Upper middle	16	0.43	0.34	0.53	4	0.41	0.27	0.55
Lower middle	7	0.63	0.57	0.69	7	0.54	0.31	0.77
Instrument								
BDI	4	0.40	0.22	0.57	–	–	–	–
BDI-II	3	0.68	0.63	0.74	–	–	–	–
CES-D	4	0.51	0.25	0.76	–	–	–	–
DASS-21	11	0.56	0.47	0.65	11	0.51	0.39	0.63
GADS	1	0.46	0.41	0.52	–	–	–	–
GHQ-28	1	0.06	0.03	0.12	–	–	–	–
HADS	2	0.10	0.06	0.13	–	–	–	–
PHQ-9	3	0.44	0.25	0.63	–	–	–	–
PSS-10	–	–	–	–	4	0.66	0.28	1.04
SRDS	1	0.26	0.24	0.29	–	–	–	–
Study quality								
Fair	13	0.47	0.36	0.58	8	0.56	0.40	0.72
Good	17	0.48	0.38	0.58	7	0.53	0.31	0.76

Note: I^2^ (Heterogeneity) of subgroup analyses was above 85%. Abbreviations: DS, Depressive Symptoms; BDI, Beck’s Depression Inventory; BDI-II, Beck’s Depression Inventory-II; CES-D, Center for Epidemiologic Studies Depression Scale; DASS-21, Depression Anxiety Stress Scale 21; GADS, Goldberg Anxiety and Depression Scale; GHQ-28, General Health Questionnaire-28; PHQ-9, Patient Health Questionnaire-9; PSS-10, Perceived Stress Scale-10, SRDS, Self-Rated-Depression-Scale.

## Data Availability

Not applicable.

## Supplementary Materials

The following supporting information can be downloaded at: https://doi.org/10.5281/zenodo.18710903 (uploaded on 20 February 2026). Table S1: Descriptive characteristics; Table S2: Quality assessment; Table S3: Factors associated with depressive symptoms; Table S4: Factors associated with stress; Figure S1: Severity level for depressive symptoms; Figure S2: Prevalence of depressive symptoms by region, income, tool, and study quality; Figure S3: Stress prevalence by severity level; Figure S4: Stress prevalence by region, income, tools, and study quality; Figure S5: Funnel plots of depressive symptoms and stress levels

## Public Involvement Statement

No public involvement in any aspect of this research.

## Guidelines and Standards Statement

This manuscript was drafted against the Preferred Reporting Items for Systematic Reviews and Meta-Analyses (PRISMA) guidelines [24]. A complete list of reporting guidelines can be accessed via the equator network: https://www.equator-network.org/reporting-guidelines/prisma/ (accessed on 6 June 2024).
